# When an uncommon condyloma revealed a multiple myeloma: Case report

**DOI:** 10.1016/j.amsu.2021.102892

**Published:** 2021-09-25

**Authors:** Sara Bouabdella, Soraya Aouali, Hanan Ragragui, Nada Zizi, Siham Dikhaye

**Affiliations:** a Department of Dermatology, Mohammed the VIth University Hospital of Oujda, Morocco; b Laboratory of Epidemiology, Clinical Research and Public Health, Faculty of Medicine and Pharmacy, Mohamed the First University of Oujda, Morocco

**Keywords:** Amylosis, Multiple myeloma, Condyloma

## Abstract

**Introduction and importance:**

Condyloma are a common and easily diagnosticated condition that could affect the area around and inside the anus. But a nodular perianal lesion is not always a simple condyloma.

**Case presentation:**

We report a case of a 61-year-old patient with nodular perianal lesions mimicking condyloma that has revealed an amylosis and a multiple myeloma.

**Clinical discussion:**

The cutaneous manifestations of amyloidosis are diverse. Perianal nodular lesions were indicative of cutaneous amyloidosis in our patient. It is imperative to screen systemic involvement for amyloidosis.

**Conclusion:**

Our case report highlights the importance of minitious physical examination because some simple lesions can hide dangerous affections.

## Highlights

• A nodular perianal lesion is not always a simple condyloma.
• Cutaneous amyloidosis could be manifested by a nodural perianal lesion.
• Some simple cutaneous lesions can hide dangerous affections.

## 1 Introduction

Condyloma are a common and easily diagnosticated condition that could affect the area around and inside the anus. Usually, it doesn't cause pain or discomfort and patients may be unaware that the warts are present. But a nodular perianal lesion is not always a simple condyloma. We report the observation of a patient with nodular perianal lesions mimicking condyloma that has revealed a multiple myeloma.

Our case report was written according to CARE guidelines [1].

## 2 Case presentation

**Fig. 1:**
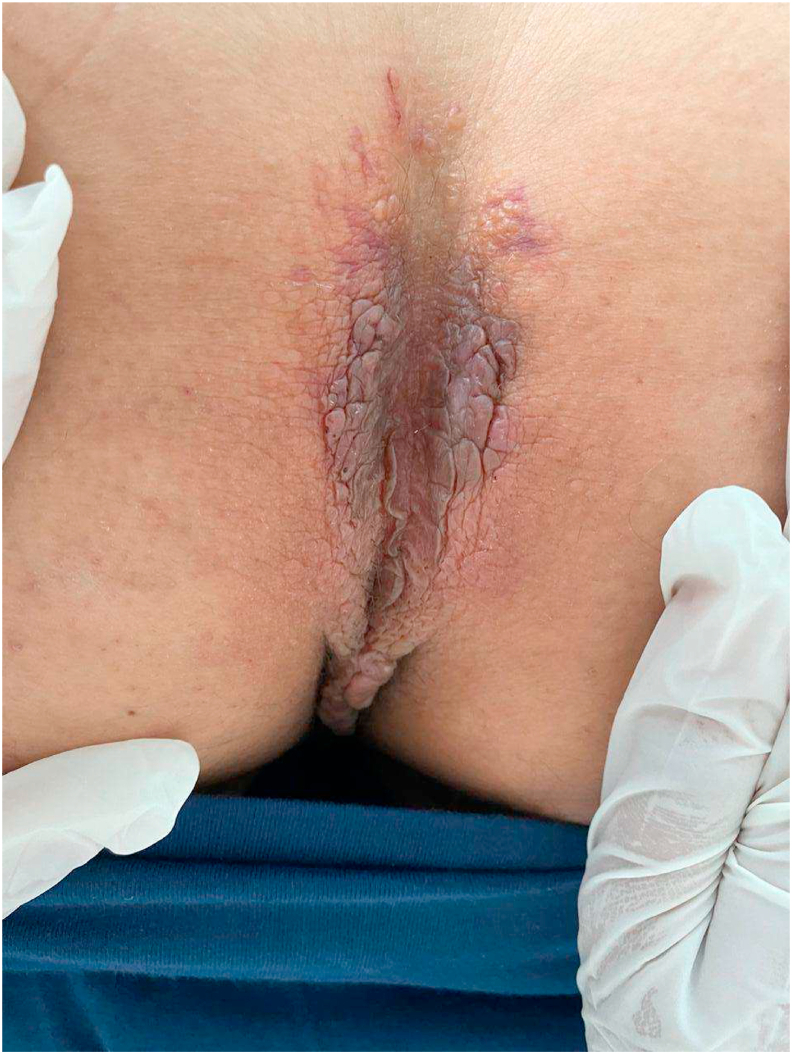
Clinica imgae: purplish nodular lesions in the intergluteal area.

**Fig. 2:**
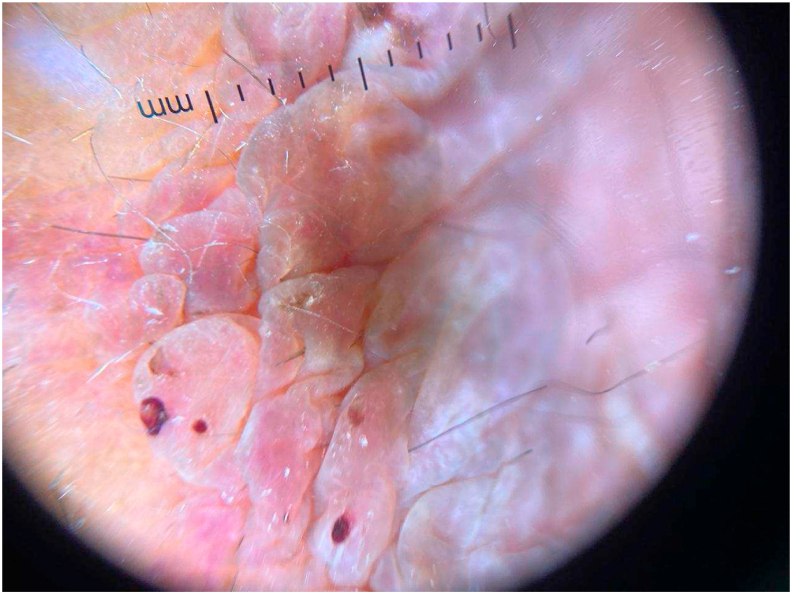
Dermoscopic image showing several thromboses and multiple papular pinkish lesions.

**Fig. 3:**
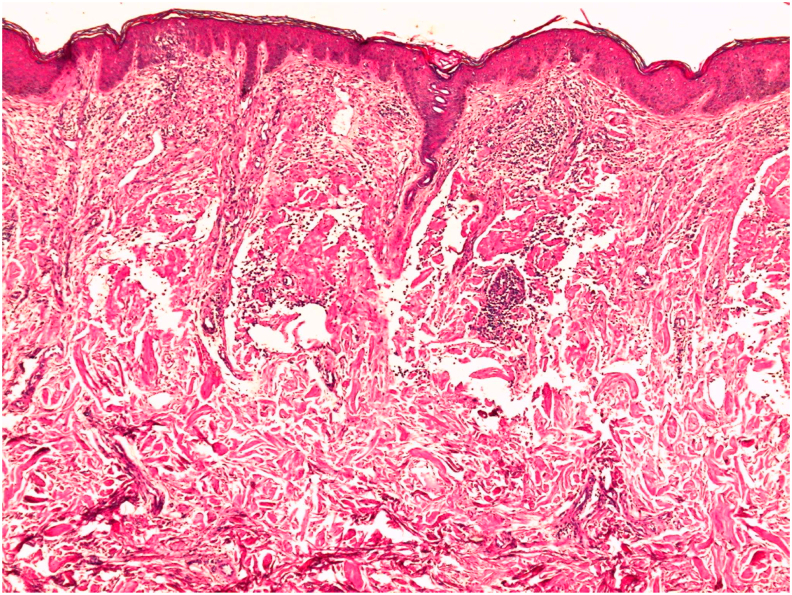
Histological image: Nodular deposits of eosinophilic dermis anhistatic and cracked + mononuclear inflammatory infiltrates (HES G stain x 50).

**Fig. 4:**
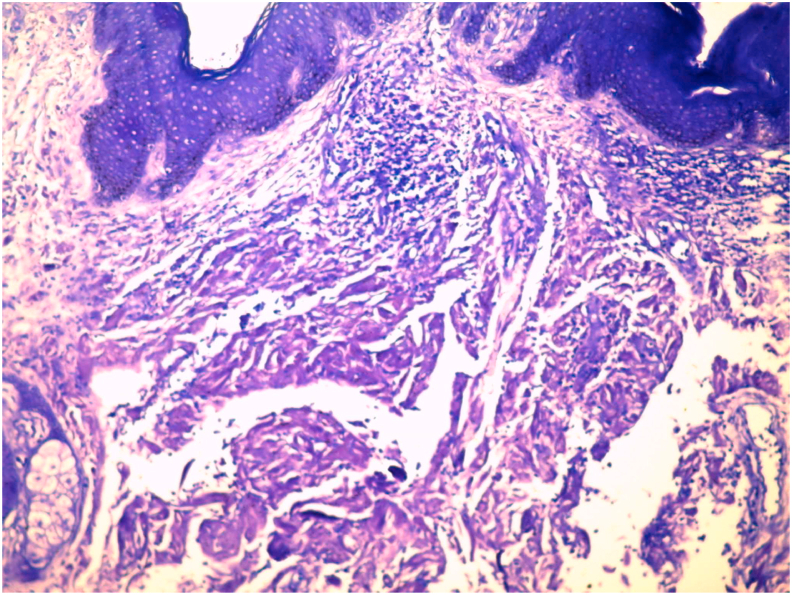
Histological image: Amyloid deposits stained in Indian pink with Cristal Violet (Cristal Violet staining G x 100).

A 61-year-old male patient, with iron deficiency anemia for 1 year treated by iron supplementation, consulted for purplish, poorly limited, non-itching and painless erythematous cupboards of the forearms and legs. The patient had no previous medical history of a similar eruption and there was no similar cases in the patient's family. Clinical examination revealed confluent, purpuric and ecchymotic plaques in the back of both hands, forearms and the right shoulder. We also noted bluish macules located on the palmar face of the 1st, 2nd and 3rd finger of the right hand and the palmar face of the 2nd left finger, with mega capillaries in the capillaroscopy. On anogenital examination, the patient has purplish nodular lesions in the intergluteal area (Fig. 1) with a several thromboses seen by dermoscopy (Fig. 2) and multiple papular lesions of flesh color and other pinkish lesions in the scrotal area. The patient hasn't organomegaly, lymphadenopathy, or auscultatory abnormality. A biopsy of the anal lesions was done and the anatomopathological study revealed papillary and especially reticular dermis invaded by nodular deposits of an anhist and cracked eosinophilic substance condensing around the vessels and adnexal structures (Fig. 3, Fig. 4). This substance was PAS positive and was enhanced by Crystal Violet and Red Congo with yellow-green dichroism in polarized light. The salivary gland biopsy revealed a discreet and chronic sialadenitis grade 1 of Chilsholm and Masson with no evidence for dry syndrome nor amyloidosis. Differential diagnoses included condyloma, lichen and Buschke-Lowenstein tumor. Further clinical evaluation showed systemic amyloidosis. Cardiovascular examination revealed irregular heart sounds without heart murmur and the EKG showed atrial fibrillation with average ventricular rate of 70 bpm, left axis, micro voltage in peripheral leads, left anterior hemiblock with monomorphic ventricular extrasystoles. A holter ECG objectified a baseline atrial flutter rhythm with sinus rhythm passages and the *trans*-thoracic ultrasound was in favor of a nonobstructive, restrictive symmetrical cardiomyopathy, most likely secondary to amyloidosis with Ejection Fraction at 57%. The electrophoresis of serum proteins showed hypoglobulinemia, hyper alpha1-globulinemia, a doubling of the alpha2-globulinemia zone with restriction of the heterogeneity of gamma globulins and serum immunofixation demonstrated the presence of light chains lambda. Urinary protein electrophoresis and Bence Jones proteinuria demonstrated a free lambda-like protein. The assay of the serum kappa and lambda free light chains revealed high figures suggesting multiple myeloma. A sternal puncture was therefore performed objectifying a 30% plasmacytosis, which confirmed the diagnosis. The biological assessment also revealed a hypercalcemia at 113 mg/l (The normal reference of serum calcium level: 4.6–5.3 g/dl). The evaluation suggested AL amyloidosis and indicated a malignant course with skin and cardiac involvement. The patient was candidate for chemotherapy and was referred to the oncology department. The patient presented to the emergency department one week later for an acute dyspnea and died.

## 3 Discussion

Amyloid is an extracellular protein material that is amorphous, eosinophilic, homogeneous, and hyaline [2]. Amyloidosis is a spectrum of diseases consisting of the deposition of amyloid proteins in various tissues [3]. Deposits may be limited in the skin with no sign of systemic involvement (primary localized cutaneous amyloidosis) or may be systemic and involve multiple organs and tissues (primary or secondary systemic amyloidosis) [4]. Cutaneous amyloidosis is subdivided into macular, papular and nodular amyloidosis [5]. Nodular amyloidosis is the rarest type and has somewhat distinct characteristics compared to the other two types [5]. Nodular amyloidosis was first described by Gottron in 1950. Its skin lesions may present as a single, or rarely, multiple nodules, sometimes covered with atrophic plaques. We also can have papules or plaques [3]. These lesions are firm, smooth, waxy or rubbery, pink to tan, measuring up to several centimeters. On some lesions, telangiectasia may be observed [6]. Bullous and anetodermal lesions have been reported [6]. It preferably affects the acral areas, followed by the legs, head, trunk, arms and genital area, respectively [5]. Our patient has both multiple nodules and purpuric macules, which is rare. Nodular cutaneous amyloidosis affects patients aged between 50 and 60 years with an average age of 55 years without sexual predilection. On the anatomopathological examination, the overlying epidermis may show atrophic changes. The entire dermis and sometimes the subcutaneous area are filled with amorphous, eosinophilic and homogeneous amyloid material. Amyloid deposits can also be found in the walls of small blood vessels and around fat cells [6]. A focal infiltrate of plasma cells is diffused through the deposits [6]. When stained with Congo red, amyloid deposits exhibit a characteristic yellow-green birefringence under polarized light [6]. Dermal amyloid L protein is derived from light chains of immunoglobulins produced by locally infiltrated plasma cells [4]. The precise cause of localized plasma cell infiltration is unknown. Although the amyloid deposits in nodular amyloidosis are composed of AL protein, as in primary systemic amyloidosis, by definition, nodular amyloidosis is limited to the skin without any systemic involvement and is generally a benign condition [6]. However, based on the known association of nodular amyloidosis with systemic diseases, systemic evaluation and a long-term follow-up evaluation should be performed [7]. Indeed, the progression of nodular amyloidosis to systemic amyloidosis has been reported in numerous articles at a rate of 7%–50% [6]. Some cases may be associated with Sjögren's syndrome, CREST syndrome (calcinosis, Raynaud's phenomenon, esophageal motility disorders, sclerodactyly and telangiectasia), dermatomyositis and diabetes mellitus [8].

Systemic types of amyloidosis include those associated with plasma cell dyscrasia, as in multiple myeloma [9]. However, only few cases of condyloma-like perianal lesions in multiple myeloma-associated amyloidosis have been reported [9,10].

Clinically condyloma like lesions could be misdiagnosed as benign lesions and finally lead to discover underlying a malignant disease.

Some lesions of cutaneous amyloidosis can be removed by surgery or laser excision if they are cosmetically disfiguring or symptomatic. Other methods have been tried to improve the appearance of the lesions, such as intralesional corticosteroids, cryotherapy, and dermabrasion, but they are generally not helpful and have a high rate of recurrence [8].

## 4 Conclusion

Nodular cutaneous amyloidosis usually manifests as a swelling nodule at acral sites. Its etiology remains unknown. The skin biopsy provides the definitive diagnosis. It is essential to carry out a systemic extension workup and to exhaustively search for lymphoproliferation. Our case report highlights the importance of minitious physical examination because some simple lesions can hide dangerous affections.

## Ethical approval

The ethical committee approval was not required give the article type (case report). However, the written consent to publish the clinical data of the patients was given and is available to check by the handling editor if needed.

## Guarantor

Sara Bouabdella.

## Sources of funding

None.

## Author contribution

S.B.: Study concept, Data collection, Data analysis, Writing the paper. S.A.: Contributor. H.R.: Contributor. N.Z.: Supervision and data validation. S.Di.: Supervision and data validation.

## Consent

Written informed consent was obtained from the patient for publication of this case report. CARE guidelines were applied for reporting this case report’ finding.

## Registration of research studies

This is not an original research project involving human participants in an interventional or an observational study but a case report. This registration is was not required.

## Provenance and peer review

Not commissioned, externally peer-reviewed.

## Declaration of competing interest

The authors declare no conflict of interest.
